# 按需糖皮质激素治疗对再生障碍性贫血p-ALG治疗相关血清病反应发生及转归的影响

**DOI:** 10.3760/cma.j.issn.0253-2727.2023.03.006

**Published:** 2023-03

**Authors:** 夏婉 杨, 康 周, 建平 李, 慧慧 樊, 文睿 杨, 蕾 叶, 园 李, 洋 李, 广新 彭, 洋 杨, 佑祯 熊, 馨 赵, 丽萍 井, 莉 张, 凤奎 张

**Affiliations:** 中国医学科学院血液病医院（中国医学科学院血液学研究所），实验血液学国家重点实验室，国家血液系统疾病临床医学研究中心，细胞生态海河实验室，天津 300020 State Key Laboratory of Experimental Hematology, National Clinical Research Center for Blood Diseases, Haihe Laboratory of Cell Ecosystem, Institute of Hematology & Blood Diseases Hospital, Chinese Academy of Medical Sciences & Peking Union Medical College, Tianjin 300020, China

**Keywords:** 贫血，再生障碍性, 血清病, 糖皮质激素, Anemia, aplastic, Serum sickness, Glucocorticoid

## Abstract

**目的:**

探讨按需糖皮质激素治疗对再生障碍性贫血（AA）应用猪抗人淋巴细胞球蛋白（p-ALG）相关血清病反应发生及转归的影响。

**方法:**

收集2019年1月至2022年1月连续就诊于中国医学科学院血液病医院贫血诊疗中心接受p-ALG治疗的AA患者资料，其中按需治疗组患者根据血清病反应发生与否及严重程度应用糖皮质激素，共35例；传统防治组患者从同期接受常规糖皮质激素防治方案的群体中依据年龄、疾病诊断分型按1∶3进行倾向性评分匹配，共纳入105例。比较两组间血清病发生率、临床表现、治疗转归及糖皮质激素用量。

**结果:**

按需治疗组和传统防治组患者的血清病发生率分别为65.7％和54.3％，差异无统计学意义（*P*＝0.237）。两组患者血清病中位发病时间相同［第12（9, 13）天对第12（10, 13）天，*P*＝0.552］；临床症状体征相似，主要表现为关节和（或）肌肉疼痛、发热及皮疹；严重程度分级均以1～2级血清病反应为主（62.8％对51.4％），仅少数3级反应（2.9％对2.9％），无4级和5级血清病反应，不同等级血清病反应在两组间分布差异无统计学意义（*P*＝0.530）；血清病中位持续时间相同［5（3, 7）d对5（3, 6）d，*P*＝0.529］，均经糖皮质激素治疗后完全缓解。在未发生血清病反应的患者中，传统防治组平均每例患者的糖皮质激素预防量为（469.48±193.57）mg（按需治疗组预防量为0）。在发生血清病反应的患者中，按需治疗组平均每例患者的糖皮质激素治疗量显著少于传统防治组［（125.91±77.70）mg对（653.90±285.56）mg，*P*<0.001］。

**结论:**

按需治疗策略可显著减少糖皮质激素用量，而并不增加血清病发生率，血清病持续时间和2级以上血清病反应发生率较传统防治组患者相近。

## Abstract (en)

**Objective:**

To investigate the effect of on-demand glucocorticoid strategy on the occurrence and outcome of porcine anti-lymphocyte globulin (p-ALG)-associated serum sickness in aplastic anemia (AA).

**Methods:**

The data of AA patients who received in the Anemia Diagnosis and Treatment Center of Haematology Hospital, CAMS & PUMC from January 2019 to January 2022 were collected. Among them, 35 patients were enrolled in the on-demand group, with the glucocorticoid strategy adjusted based on the occurrence and severity of serum sickness; 105 patients were recruited in the usual group by matching the age and disease diagnosis according to 1∶3 ratio in patients who received a conventional glucocorticoid strategy in the same period. The incidences, clinical manifestations, treatment outcomes of serum sickness, and glucocorticoid dosage between the two groups were analyzed.

**Results:**

The incidences of serum sickness in the on-demand group and the usual group were 65.7％ and 54.3％ (*P*＝0.237), respectively. The median onset of serum sickness was the same [12 (9, 13) d *vs* the 12 (10, 13) d, *P*＝0.552], and clinical symptoms and signs, primarily joint, and/or muscle pain, fever, and rash were similar. Severity grades were both dominated by Grades 1–2 (62.8％ *vs* 51.4％), with only a few Grade 3 (2.9％ *vs* 2.9％), and no Grades 4–5. No significant difference in the serum sickness distribution (*P*＝0.530). The median duration of serum sickness was the same [5 (3, 7) d *vs* 5 (3, 6) d, *P*＝0.529], and all patients were completely cured after glucocorticoid therapy. In patients without serum sickness, the average dosage of prophylactic glucocorticoid per patient in the usual group was (469.48 ±193.57) mg (0 in the on-demand group). When compared to the usual group, the average therapeutic glucocorticoid dosage per patient in the on-demand group was significantly lower [(125.91±77.70) mg *vs* (653.90±285.56) mg, *P*<0.001].

**Conclusion:**

In comparison to the usual glucocorticoid strategy, the on-demand treatment strategy could significantly reduce glucocorticoid dosage without increasing the incidence of serum sickness; in addition, the duration of serum sickness and the incidence of above Grade 2-serum sickness were similar.

抗胸腺细胞球蛋白（ATG）和环孢素A（CsA）的联合免疫抑制治疗（IST）是不适于造血干细胞移植治疗再生障碍性贫血（AA）患者的标准一线治疗方案[1]。作为异种免疫球蛋白，传统上ATG输注期间须同步应用糖皮质激素预防即刻过敏反应，输注结束后仍需继续使用至少2～3周以预防血清病反应。在此预防基础上，一旦出现血清病，则需追加糖皮质激素用量[1]。猪抗人淋巴细胞球蛋白（p-ALG）的疗效与兔/马ATG相近[2]–[4]，近几年在国内普遍应用，预防和治疗血清病时糖皮质激素的使用仍然沿用了和兔/马ATG一致的方案。但该方案主要基于理论和经验，并无相关前瞻性研究证据支持。长时程的糖皮质激素可能导致药物相关并发症（如糖尿病、高血压、骨病、感染等）发生率增加，同时也会进一步加重AA患者骨髓的脂肪化[5]。因此减少传统方案中糖皮质激素的用量既符合临床需求，也是个体化治疗的要求。我们采取按需治疗的方案，包括不预防性应用糖皮质激素和对发生血清病反应者根据临床表现分级调整剂量，探索这一策略对AA患者p-ALG相关血清病反应发生及转归的影响，以期了解糖皮质激素的实际预防作用并优化用药方案。

## 病例与方法

1. 病例选择：本研究为回顾性队列研究。收集2019年1月至2022年1月间连续就诊于我院贫血诊疗中心接受IST方案（p-ALG联合CsA）的AA患者337例，其中35例患者根据血清病反应发生与否及严重程度采取按需糖皮质激素治疗方案，其余302例患者采用传统的糖皮质激素防治方案。对按需治疗患者及传统防治患者进行倾向性评分匹配，匹配依据为患者的年龄、疾病诊断分型，设定按需治疗组∶传统防治组为1∶3、卡钳值为0.02，最终共纳入按需治疗组患者35例，传统防治组患者105例。两组患者匹配前后变量特征详见表1。

**表1:** 按需治疗组与传统防治组再生障碍性贫血患者倾向性评分匹配前后的临床特征比较

指标	匹配前				匹配后			
	按需治疗组（35例）	传统防治组（302例）	统计量	*P*值	按需治疗组（35例）	传统防治组（105例）	统计量	*P*值
年龄[岁，*M*（*IQR*）]	43（26, 55）	34.5（24, 52）	*z*=−1.083	0.279	43（26, 55）	44（28, 56）	*z*=−0.337	0.736
疾病诊断分型[例（%）]			*χ*^2^=6.014	0.049			*χ*^2^=0.948	0.622
TD-NSAA	8（22.9）	64（21.2）			8（22.9）	26（24.8）		
SAA	24（68.6）	156（51.7）			24（68.6）	64（61.0）		
VSAA	3（8.5）	82（27.2）			3（8.5）	15（14.3）		

注 TD-NSAA：输血依赖非重型再生障碍性贫血；SAA：重型再生障碍性贫血；VSAA：极重型再生障碍性贫血

2. 血清病防治方案：两组患者接受p-ALG（武汉生物制品研究所产品）20 mg·kg^−1^·d^−1^静脉输注，同时给予等效泼尼松1 mg·kg^−1^·d^−1^剂量的糖皮质激素预防即刻过敏反应，连用5 d。CsA则以3～5 mg·kg^−1^·d^−1^分两次口服，维持谷浓度150～250 µg/L。传统防治组方案：p-ALG结束后继续口服等效泼尼松1 mg·kg^−1^·d^−1^剂量的糖皮质激素，从第15天开始减量，至第22天停药，用以预防血清病反应。当发生血清病反应时，追加等效泼尼松1 mg·kg^−1^·d^−1^剂量的糖皮质激素静脉滴注，症状缓解后停药。按需治疗组方案：p-ALG结束后无糖皮质激素预防用药，如发生血清病反应，则予静脉输注糖皮质激素，1～2级血清病反应患者给予泼尼松0.5 mg·kg^−1^·d^−1^等效剂量，3级及以上患者给予泼尼松1 mg·kg^−1^·d^−1^等效剂量，症状缓解后停药。

3. 血清病诊断及严重程度评估：血清病诊断和严重程度分级主要根据患者的临床表现进行评估，参照文献[6]–[7]标准，分级如下：1级：发热（<38.5 °C）、轻微关节或肌肉疼痛、局限性皮疹；2级：中等程度关节或肌肉疼痛，发热（≥38.5 °C）、多部位皮疹或荨麻疹；3级：严重的多发性关节或肌肉疼痛，全身广泛性皮疹；4级：危及生命的症状；5级：死亡。

4. 相关定义：血清病发生率定义为在p-ALG治疗28 d内，发生血清病反应的患者人数占所在治疗组总人数的比例。血清病发生时间是从p-ALG起始治疗时间开始计算。血清病持续时间定义为从开始发生血清病反应至症状完全缓解的间期。糖皮质激素累积量定义为从患者完成5 d p-ALG治疗，即第6天始，至糖皮质激素结束期间的总用量（按照泼尼松剂量计，下同）的总和，其中糖皮质激素预防量定义为第6天至第22天期间用于预防血清病反应的糖皮质激素用量总和，糖皮质激素治疗量定义为从患者出现血清病反应当天始，至血清病反应完全缓解日期间全部糖皮质激素用量的总和。

5. 统计学处理：采用SPSS 22.0进行统计学分析，符合正态分布的计量资料，用均值±标准差描述，组间比较采用*t*检验，非正态分布的连续变量，用中位数（*IQR*）描述，组间比较采用Mann-Whitney *U*检验，分类变量用例数（百分比）描述，组间比较采用*χ*^2^检验或Fisher精确概率法。*P*<0.05为差异有统计学意义。

## 结果

1. 一般临床特征：共纳入按需治疗组和传统防治组140例患者，男77例，女63例，男女比为1.22∶1，中位年龄44（14～71）岁。其中，输血依赖非重型AA（TD-NSAA）34例（24.3％），重型AA（SAA）88例（62.9％），极重型AA（VSAA）18例（12.9％）；初诊治疗时伴感染者27例（19.3％），不伴感染者113例（80.7％）。两组患者性别、中性粒细胞绝对计数（ANC）、HGB、PLT、网织红细胞绝对值（ARC）、治疗时有无感染、体重的差异均无统计学意义（均*P*>0.05）（表2）。所有患者按IST方案完成5 d p-ALG输注，均无严重即刻过敏反应发生。

2. 血清病反应的发生：按需治疗组患者的血清病发生率为65.7％（23/35），中位发生时间为治疗第12（9, 13）天，其中两周内发病者22例（95.6％），超过两周发病者1例（4.4％）。传统防治组患者的血清病发生率为54.3％（57/105），中位发生时间为治疗第12（10, 13）天，其中两周内发病者51例（89.5％），超过两周发病者6例（10.5％）。比较两组患者的血清病发生率和发生时间，差异均无统计学意义（*P*值分别为0.237、0.552）。

3. 血清病表现及严重程度：按需治疗组23例发生血清病反应的患者中9例（39.1％）发热，8例（34.8％）出现皮疹，17例（73.9％）出现关节和（或）肌肉疼痛，1例（4.4％）表现为胸腔积液。传统防治组57例发生血清病反应的患者中27例（47.4％）发热，17例（29.8％）出现皮疹，43例（75.4％）出现关节和（或）肌肉疼痛，8例（14.0％）出现不同程度的腹痛、胸痛、肝肾功能异常等。两组患者出现不同临床表现的构成差异均无统计学意义（均*P*>0.05）。

按需治疗组患者中，1～2级血清病反应占62.8％（22/35），3级血清病反应占2.9％（1/35）。传统防治组中，51.4％（54/105）的患者为1～2级血清病反应，2.9％（3/105）的患者为3级血清病反应。两组均无4级和5级血清病反应。不同等级血清病反应在两组间分布差异无统计学意义（*P*＝0.530）。

4. 血清病的转归：按需治疗组患者的中位血清病持续时间为5（3，7）d，其中2例（8.7％）血清病持续时间为1 d（发病当天应用糖皮质激素治疗即可获得缓解），18例（78.3％）血清病症状在1周内缓解，最长血清病持续时间为18 d。传统防治组患者的中位血清病持续时间为5（3，6）d，其中有3例（5.3％）糖皮质激素治疗当天症状缓解，47例（82.5％）于1周内症状好转，最长持续时间为14 d。两组患者的血清病持续时间差异无统计学意义（*P*＝0.529）（图1）。两组患者的血清病反应症状均得到完全缓解，未观察到不可逆转损伤。

**表2:** 按需治疗组与倾向性评分匹配后传统防治组再生障碍性贫血患者的临床特征比较

指标	按需治疗组（35例）	传统防治组（105例）	统计量	*P*值
性别［例（％）］			*χ^2^*=0.779	0.377
男	17（48.6）	60（57.1）		
女	18（51.4）	45（42.9）		
ANC［×10^9^/L，*M*（*IQR*）］	0.51（0.40, 0.90）	0.48（0.28, 0.75）	*z*=−0.915	0.132
HGB［g/L，*M*（*IQR*）］	67（58, 81）	66（58, 76）	*z*=−0.915	0.360
PLT［×10^9^/L，*M*（*IQR*）］	12（7, 21）	12（7, 17）	*z*=−0.684	0.494
ARC［×10^12^/L，*M*（*IQR*）］	0.020（0.013, 0.044）	0.020（0.010, 0.040）	*z*=−0.664	0.506
治疗时有无感染［例（％）］			*χ^2^*=0.015	0.902
有	7（20.0）	20（19.0）		
无	28（80.0）	85（81.0）		
体重［kg，*M*（*IQR*）］	68（59, 77）	69（56, 78）	*z*=−0.833	0.405

注 ANC：中性粒细胞绝对计数；ARC：网织红细胞绝对值

**图1:**
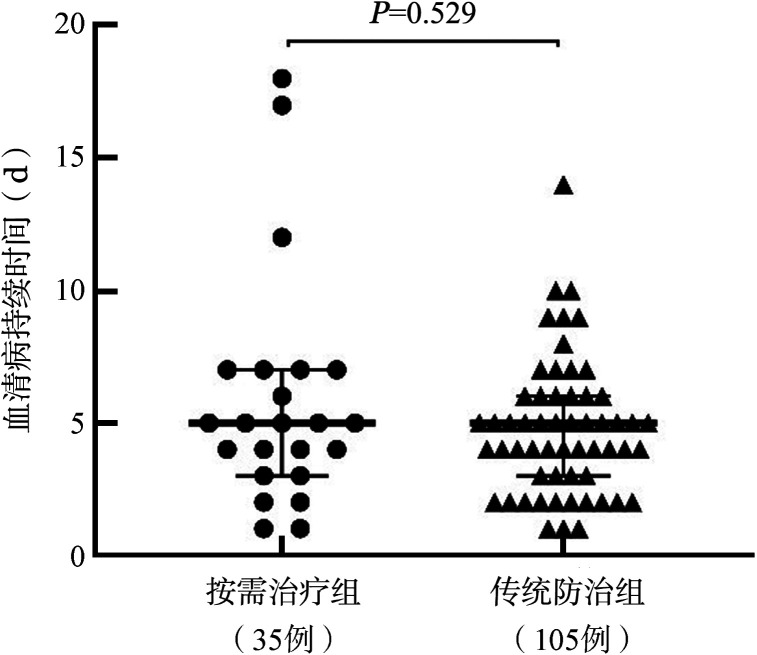
按需治疗组和传统防治组再生障碍性贫血患者的血清病持续时间

5. 糖皮质激素用量：所有患者的糖皮质激素用量均按照泼尼松剂量进行计算。按需治疗组平均每例患者的糖皮质激素累积量显著少于传统防治组［（82.74±87.08）mg对（569.59±263.37）mg，*P*<0.001］。在未发生血清病反应的患者中，传统防治组平均每例患者的糖皮质激素预防量为（469.48±193.57）mg，按需治疗组预防量为0 mg。在发生血清病反应的患者中，按需治疗组平均每例患者的糖皮质激素治疗量亦显著少于传统防治组［（125.91±77.70）mg对（653.90±285.56）mg，*P*<0.001］。

## 讨论

血清病的概念由von Pirquet和Schick于1905年首次提出，是一种因异种血清制品进入体内产生血循环免疫复合物而引起的Ⅲ型变态反应性疾病[8]。国内报道的ATG/ALG相关血清病发生率为52.0％～68.0％[9]–[12]。本研究中，按需治疗组和传统防治组患者的血清病发生率分别为65.7％和54.3％，与文献报道相近，且两组相比差异无统计学意义（*P*＝0.237），提示不预防性应用糖皮质激素治疗并未增加血清病发生率。进一步分析两组中发生血清病反应患者的临床表现，血清病反应中位发生时间均为第12天，多数患者的发病时间集中在p-ALG治疗两周内（95.6％对89.5％，*P*＝0.552）；其症状体征相似，主要表现为关节和（或）肌肉疼痛、发热、皮疹，少见腹痛、胸痛、肝肾功能异常、体腔积液等不典型症状。以上结果显示是否给与预防性糖皮质激素不会改变血清病反应的发生时间和表现形式。两组患者的血清病反应均以1～2级的轻型血清病表现为主，仅少数3级反应（2.9％对2.9％），无 4级和5级血清病反应。尽管血清病反应严重程度在两组间分布差异无统计学意义（*P*＝0.530），但按需治疗组的轻症血清病反应发生率（62.8％）略高于传统防治组（51.4％），仍可能具有现实意义，我们推测预防性糖皮质激素的应用不会降低重症血清病反应的发生，可能仅是减少了轻症患者的发生。

糖皮质激素治疗血清病反应的转归包括临床表现的缓解速度和缓解质量。研究显示，按需治疗组与传统防治组发生血清病反应的患者的持续时间相近［5（3, 7）d对5（3, 6）d，*P*＝0.529］，大多数患者的血清病反应都可在1周内好转（78.3％对82.5％），表明按需给药策略不会影响血清病反应的总时程，同时我们也观察到两组患者的血清病反应症状均能得到完全缓解，无不可逆转损伤发生。

糖皮质激素是ATG/ALG治疗方案中的重要辅助性药物，但也会引起诸多不良反应发生[13]。此外对于接受IST的AA患者，由于细胞免疫受抑制，本身就存在较高的感染风险[14]–[15]，因此，减少IST期间不必要糖皮质激素的使用尤为重要。本研究结果显示，在未发生血清病反应的患者中，按需治疗组每例患者的平均糖皮质激素预防剂量（0 mg）较传统防治组的（469.48±193.57）mg形成鲜明对照。即使出现血清病反应，我们及时发现、及时治疗，根据不同级别的严重程度采取相应的按需糖皮质激素方案，也能显著减少糖皮质激素的治疗用量［（125.91±77.70）mg对（653.90±285.56）mg，*P*<0.001］。这些均表明按需治疗策略能够避免糖皮质激素的过量使用，从而减少相应并发症的出现概率。

总之，按需治疗策略可显著减少糖皮质激素用量，而并不增加血清病发生率，重型血清病发生率和血清病持续时间较传统防治组患者相比并无差别，支持p-ALG相关血清病的预防和治疗采用按需糖皮质激素方案。

本研究为回顾性观察研究，且纳入观察的部分病例随访时间较短，没有对长期糖皮质激素相关不良反应及AA疗效的影响做进一步深入研究，存在一定的局限性。此外，本研究纳入的患者均接受p-ALG治疗，研究结果是否同样适用于其他动物来源ATG制剂也需进一步探讨。
